# DFMD: Fast and Effective DelPhiForce Steered Molecular Dynamics Approach to Model Ligand Approach Toward a Receptor: Application to Spermine Synthase Enzyme

**DOI:** 10.3389/fmolb.2019.00074

**Published:** 2019-09-04

**Authors:** Yunhui Peng, Ye Yang, Lin Li, Zhe Jia, Weiguo Cao, Emil Alexov

**Affiliations:** ^1^Computational Biophysics and Bioinformatics Lab, Department of Physics, Clemson University, Clemson, SC, United States; ^2^Department of Genetics and Biochemistry, Clemson University, Clemson, SC, United States; ^3^Department of Physics, University of Texas, El Paso, TX, United States

**Keywords:** electrostatic interaction, steered molecular dynamics simulation, protein-ligand binding, spermine synthase, electrostatic funnel, electrostatic forces

## Abstract

Here we report a novel approach, the DelPhiForce Molecular Dynamics (DFMD) method, for steered molecular dynamics simulations to model receptor-ligand association involving charged species. The main purpose of developing DFMD is to simulate ligand's trajectory toward the receptor and thus to predict the “entrance” of the binding pocket and conformational changes associated with the binding. We demonstrate that the DFMD is superior compared with molecular dynamics simulations applying standard cut-offs, provides correct binding forces, allows for modeling the ligand approach at long distances and thus guides the ligand toward the correct binding spot, and it is very fast (frequently the binding is completed in <1 ns). The DFMD is applied to model the binding of two ligands to a receptor (spermine synthase) and it is demonstrated that it guides the ligands toward the corresponding pockets despite of the initial ligand's position with respect to the receptor. Predicted conformational changes and the order of ligand binding are experimentally verified.

## Introduction

Electrostatic interactions play critical role in wide range of biological phenomena, including receptor-ligand recognition (Honig and Nicholls, 1995; McCammon, 2009). One of the main obstacles of modeling long-range macromolecular interactions is the presence of water phase, which requires methods for correct calculations of forces in such inhomogeneous environment (Norberg and Nilsson, 2000; Yang et al., 2016). While molecular dynamics (MD) simulation with explicit water model is a popular method to study dynamics of biomacromolecules (Brooks et al., 1983; Pearlman et al., 1995; Phillips et al., 2005; Hess et al., 2008), it is computationally too demanding and non-effective in simulating ligand approach toward the receptor. To reduce computational cost associated with explicit water modeling, one applies Generalized Born (GB) models to compute electrostatic solvation energy and screening effects (Onufriev et al., 2002; Feig et al., 2004; Mongan et al., 2007). However, the GB calculations can be also quite computationally costly, especially if the system is made of large biomolecule(s). Thus, one applies a cut-off for both the effective Born radii calculations and for pair-wise interactions to speed up the simulations. Such a tradeoff is acceptable in many cases, however, if one wants to model ligand's approach toward receptor (especially if the initial position of the ligand is farther way from the receptor than the cut-offs), it will result in omitting important effects (forces) guiding the ligand toward the receptor. This prompted the development of a tool, the DelPhiForce (Li et al., 2017a) steered Molecular Dynamics (DFMD) simulations, which we report in this paper. The primary application areas of the DFMD are interactions involving charged ligands.

It should be clarified that the main goal of this development is to enable fast simulations of ligand's trajectory toward the receptor, especially in cases where the entrance of the binding pocket is unknown. This requires the ligand to be positioned far away from the receptor and at different positions so to explore various plausible trajectories. This is specifically important if the ligand is highly charged, since if the ligand has large net charge and it is positioned close to the surface of the receptor, it tends to bind to the closest surface patch with an opposite polarity. However, if such a ligand is positioned far away, then the corresponding guiding forces guide it to the entrance of the binding pocket, and thus allows the ligand to follow “correct” binding trajectory.

In this work, we apply DFMD on a particular receptor-ligands case, the spermine synthase (SpmSyn) that binds two ligands: spermidine and AdoMet (Pegg and Michael, 2010). Our interest in this enzyme is that mutations (Zhang et al., 2010; Peng et al., 2016) in SpmSyn are associated with a severe disease, the Snyder-Robinson syndrome (Albert et al., 2013), and very little is known about how the ligands bind to SpmSyn. There is an experimental structure of SpmSyn with ligands bound (Wu et al., 2008), but both ligands are buried, and it is not clear how the ligands get inside the binding pockets, a question that we address in this paper. In addition, it was shown experimentally that SpmSyn function only as a homo-dimer (Wu et al., 2008), and we address this observation via DFMD simulations as well. Last, the SmpSyn binds two ligands but it is not clear if they bind independently or in sequential other—in this work we show that they inhibit each other and speculate that they either bind almost simultaneously or AdoMet binds first followed by spermidine. To further strengthen the computational findings, we carried experimental measurements to confirm the computational predictions.

Before proceeding with the rest of the paper, we would like to clarify that we do not question the fundaments of GB formalism. Indeed, it was repeatedly shown in the literature that GB and Poisson-Boltzmann (PB) deliver energies and forces that are quite similar to each other (Feig et al., 2004; Wagoner and Baker, 2006; Anandakrishnan et al., 2011; Fogolari et al., 2013, 2015; Tolokh et al., 2018). However, the default implementation of GB models with cut-offs (in order to assure computational efficiency) results in inefficient modeling of ligand's trajectories, while removing the cut-offs makes the simulations extremely slow. The DFMD offers efficient alternative. Furthermore, it should be clarified that DFMD differs from Self-Guided MD (SGMD) method developed by BR Brooks and co-authors (Wu et al., 2013, 2016). The SGMD is aimed at enhancing conformational search efficiency through acceleration of low frequency motions present in the molecular system of interest. This is done by adding a guiding force into the equation of motion, but this force is calculated based on a local average of MD delivered momentums and forces, in contrast with DFMD, where forces are calculated independently of the MD protocol. The DFMD is similar to the approach reported by Rocchia et al. where adaptive electrostatic bias was applied to drive receptor-ligand binding by applying screened Coulombic potential (Spitaleri et al., 2018). Other researchers also focused on revealing the binding processes, and a particular example is the work by Chong et al. utilizing weighted ensemble path sampling strategy to orchestrate molecular dynamics simulations (Zwier et al., 2016). Some of these efforts resulted in software for modeling association receptor-ligand such as HTMD (Doerr et al., 2016, 2017) and SEEKR (Votapka et al., 2017). The DFMD is simply alternative protocol targeting mostly case involving charged ligands being attracted electrostatically to the receptor, which a particular emphasis when the ligand is far away from the receptor.

It should be mentioned that in the past the usage of PB in MD simulations was a subject of many investigations and developments. The most extensive work was done by Luo et al. with a main focus to provide accurate assessment of solvation energy while avoiding sharp changes of the force resulting from change of atomic positions at the solute-solvent interface (dielectric border) (Wang et al., 2009, 2010). The problems arising from interface conditions were targeted by the immerse interface method and by removing charge singularity (Wang et al., 2009). They reported successful folding of betabetaalpha1 and villin headpiece by molecular dynamics with the PB implicit solvent applying self-guiding forces (Wen et al., 2004). The problem of applying PB in MD was tackled by Wei and co-workers as well and they reported a new formulation of electrostatic forces to avoid artifacts arising from sharp molecular surfaces. It was done by directly differentiating the electrostatic potential. Furthermore, dielectric boundary forces were evaluated at the solvent–solute interface using an accurate Cartesian-grid surface integration method (Geng and Wei, 2011). In the abovementioned cases, the researchers were mostly focusing on testing the possibility of using PB formalism to deliver solvation energy and the corresponding forces within the macromolecule of interest. In contrast, the DFMD aims at delivering electrostatic forces generated by a receptor on the corresponding ligand. As mentioned above, in most of the time this is done when the ligand is far away from the receptor, so the forces are relatively small and are not critically affected by changes in the dielectric boundary solute-solvent.

## Results and Discussion

This section is organized as follows: First, we describe the DFMD protocol; second, test DFMD on SpmSyn-spermidine/AdoMet binding and compare with MD results; third, deliver predictions about ligand trajectories and importance of SpmSyn homo-dimerization, the “entrance” of the corresponding binding pocket and associated conformational changes and provide experimental verification for the computational findings.

### Overall Methodology of DelPhiForce MD Protocol

**Figure 1:**
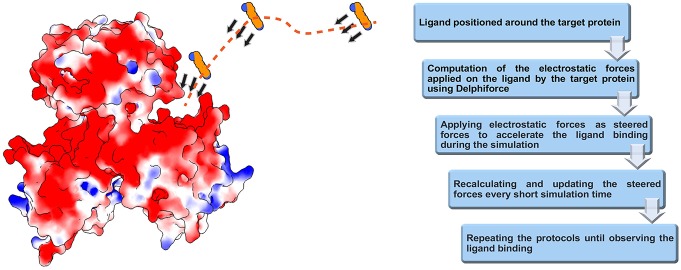
Schematic representation of DFMD algorithm. Left panel: The receptor, the SpmSyn, is shown with surface electrostatic potential (blue for positive potential while red for negative potential) and the ligand, the spermine, in ball presentation. Three representative positions over the trajectory of spermidine are shown and with arrows, we indicate the DelPhiForces assigned to each atom. The trajectory is shown with a dotted curve. Right panel: The workflow of the algorithm.

We developed DelPhiForce steered molecular dynamics (DFMD) approach which enables fast and accurate modeling of receptor-ligand binding process. The DFMD method combines accurate long-range electrostatic force calculations via DelPhiForce with a major molecular dynamics simulation package NAMD (Phillips et al., 2005). As a result, the DFMD delivers correct electrostatic forces and applies them in the MD simulations resulting in fast and accurate binding protocol. The main idea of DFMD simulation for modeling receptor-ligand binding is briefly described in Figure 1 (details are provided in method section). The ligand is placed at a distance away from the receptor assuring that the only non-zero force is the long-range electrostatic force (as outlined in the introduction, the ligand must be placed away from the receptor to avoid unwanted local effects). Then, the electrostatic forces acting on each atom of the ligand due to the charges of the receptor are computed with DelPhiForce (Li et al., 2017a,b). The forces are given to steered MD module of NAMD and short steered MD is carried out. A new position and orientation of the ligand are subjected to DelPhiForce to update the atomic forces and iterations are then repeated (Figure 1). Depending on the goals of the modeling, the user is given three options: (a) accelerated modeling, such that the atomic DelPhiForces are calculated constantly until the end of simulation; (b) cut-off modeling, such that the atomic DelPhiForces are calculated only if all ligand atoms are farther away from the receptor than the cut-offs in MD simulations; and (c) scaled modeling, such that the atomic DelPhiForces are corrected for the forces calculated via GB (the third option guarantees that there is no double-counting of electrostatic forces in MD).

### Test of DFMD and Comparison With Implicit Solvent Standard MD

It is carried out on spermine synthase (SpmSyn) receptor binding two types of charged ligands, spermidine (net charge +3e) and AdoMet (net charge +1e) (Wu et al., 2008). The reason for selecting SpmSyn for benchmarking DFMD is that the binding involves charged ligands, so the electrostatics is expected to be an important factor.

**Figure 2:**
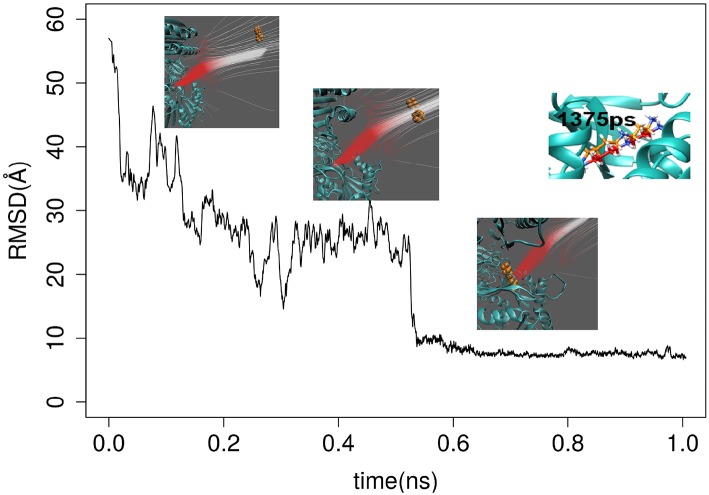
RMSD of spermidine over the time as it approaches the binding pocket of SpmSyn homo-dimer. Three representative snap shots are show along with the electrostatic field lines around the ligand. The upper-right panel shows the superimposition of the X-ray position of the SPD and a snapshot at 1,375 ps.

The first round of testing was done by applying both GBIS MD and DFMD simulations to model spermidine binding to SpmSyn, starting from spermidine positioned 60Å away from the SpmSyn (as mentioned above, the ligand should be positioned away from the receptor to allow the electrostatics to guide the ligand to the correct binding spot and thus avoiding binding to local surface patches of an opposite polarity). Thus, we carried three 100 ns simulation with GBIS MD using standard cut-off of 18 Å (both for Born radii calculations and pair-wise interactions) and the ligand showed no tendency to bind to SpmSyn (Supplementary Video 1). Then, we increased both cut-offs to 120 Å and noticed that the ligand makes more contacts with SpmSyn but still is not able to bind into the pocket (Supplementary Video 2). In contrast, applying a short DFMD, the substrate successfully steered into the binding pocket of SpmSyn in <0.5 ns simulation time (Supplementary Video 3). The success of the binding was evaluated by computing RMSD of spermidine. It was done by aligning the simulation frames (heavy atoms only) to the experimental SpmSyn homo-dimer structure with bound spermidine (Figure 2). It can be seen that DFMD simulation reproduces highly accurately binding position resulting in RMSD of 5 Å with respect to experimental structure. This indicates two things: DFMD is successful in modeling the binding while GBIS MD is not and at the same time, the DFMD is very fast bringing the ligand from 60 Å away to the binding pocket in 0.5 ns.

These observations prompted us to investigate the electrostatic forces computed via GBIS (with cut-offs 120 Å) and DelPhiForce. First, we tested the sensitivity of GBIS delivered electrostatic forces on a “toy” system made of SpmSyn and two ions (one ion providing the electrostatic potential and another receiving it—see Supplementary Material). Keeping the distance between ions the same, in one case we placed the SpmSyn away from ions, in another case, between the ions (Figure S1). One expects that the electrostatic force on the receiving ion should be different in these two cases. However, GBIS forces are practically the same (Table S1), while DelPhiForce calculations resulted in different forces as to be expected (Table S1). We further repeated the calculations by replacing the receiving ion with spermidine and then compared the forces in these two scenarios. Similar tendency was observed that GBIS forces are almost identical in these two cases while DelPhiForces are not (Figure S2). Another comparison was made on the receptor-ligand studied in this work. Thus, the ligand, the spermidine, was positioned 60 Å away from SpmSyn along X and Y directions, and then we computed the forces with GBIS and DelPhiForce (Figure S3). The results are shown in Figure S4, and one can see that the forces are different with a tendency DelPhiForces to be about 3 times larger than forces delivered by GBIS. Perhaps this is the reason why DFMD was successful of guiding the spermidine to the binding pocket of SpmSyn, while GBIS MD was not.

### Modeling Spermidine and AdoMet Association With SpmSyn

#### The Effect of Selecting Initial Position of the Ligand

**Figure 3:**
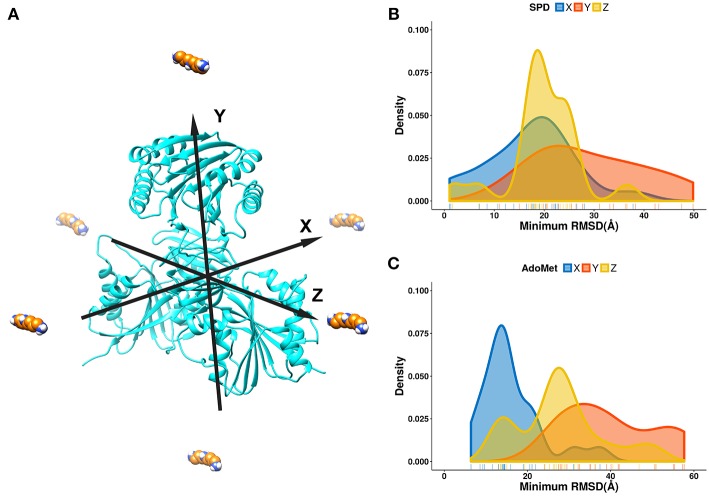
DFMD results for AdoMet and spermidine at different initial positions. **(A)** Six different initial positions were generated, where the ligand is moved along X, -X, Y, -Y or Z, -Z axes 60 Å away from the geometrical center of SpmSyn. **(B,C)** Distribution of the minimal RMSD of the ligands (SPD—spermidine; AdoMet) over 10 trajectories.

For the purpose of this investigation, the ligands, spermidine and AdoMet, were positioned 60 Å away from the geometrical center of SpmSyn along each of the axes (X, Y, and Z) (Figure 3A). The goal is to probe if the ligands starting from quite different positions will be able to find their way inside the corresponding binding pocket. Ten independent simulations were performed and then the RMSD of the substrates were computed via alignment of the simulation frames to the experimental bound structure using heavy atoms only. The minimal RMSD value from each simulation run was taken to derive the probability density map (representing of the best substrate position with respect to the experimental one over ten independent runs) (Figures 3B,C). We observe that the success rate depends on the initial position of the ligands: the highest success is seen if the ligand is positioned toward the corresponding binding pocket. However, it is very encouraging that we observe successful binding even for initial positions being at the top or the bottom of SpmSyn, clearly far away from the corresponding binding pockets. The success is more prominent for highly charged spermidine (+3e) and less impressive for AdoMet (+1e).

#### Role of Homo-Dimerization

**Figure 4:**
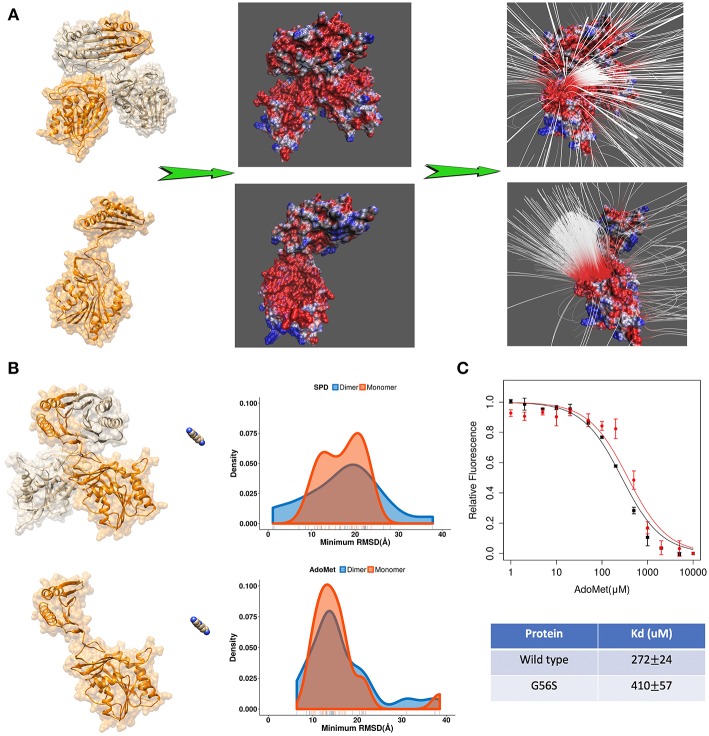
**(A)** The electrostatics (left—the electrostatic potential mapped on the surface, right—the electrostatic field lines) of spermine synthase homo-dimer and monomer. **(B)** Comparison of the density map (minimum RMSD) for substrates in case of spermine synthase homo-dimer and monomer. **(C)** Experimentally measured relative florescence of wild-type spermine synthase and mutant spermine synthase binding AdoMet and the corresponding dissociation constant (Kd).

Further we applied DFMD to provide an explanation of the role of homo-dimerization of SpmSyn. It is experimentally shown that SpmSyn functions only as a homo-dimer, despite that each monomer has well-defined active pockets (Peng et al., 2016). In the past, we speculated that this is due to the formation of electrostatic funnel caused by the dimerization that guides the ligands to the active site. Indeed, electrostatic potential calculations with DelPhi and electrostatic field lines visualized with VMD (Figure 4A) show that the electrostatic funnel is well-formed in case of SpmSyn homo-dimer and becomes much wider in the monomer, and thus does not provide specific guidance of the ligands toward the binding pockets. In the DFMD simulations, ligands were positioned 60 Å along X-axis in respect to either the dimer or the monomer. Ten independent simulations were performed and the smallest RMSD value from each simulation run was taken to derive the probability density map (representing of the best substrate position with respect to the experimental one over ten independent runs) (Figures 3B,C). Comparing the results in case of homo-dimer and monomeric SpmSyn (Figure 4B), one can see that the success rate is much more pronounced for the homo-dimer. Such an observation is consistent with the electrostatic analysis provided above that the electrostatic funnel is much better formed in the homo-dimer compared with the monomer (Figure 4A). Comparing the results for AdoMet (+1e) and spermidine (+3e), one can see that the dimerization affects highly charged substrate (spermidine) more than the less charged AdoMet. Relative florescence experiments were performed to validate our computational results (Figure 4C). We selected a mutation, G56S mutation, which was previously shown to prevent dimerization while having no effect on protein stability (Peng et al., 2016). The experimental measurements confirmed that the AdoMet disassociation constant (Kd) is smaller in case of homo-dimer comparing with the monomer (Figure 4C), which is expected to result in a stronger binding of AdoMet to SpmSyn homo-dimer.

#### Predicting the “Entrance” of the Corresponding Binding Pockets

**Figure 5:**
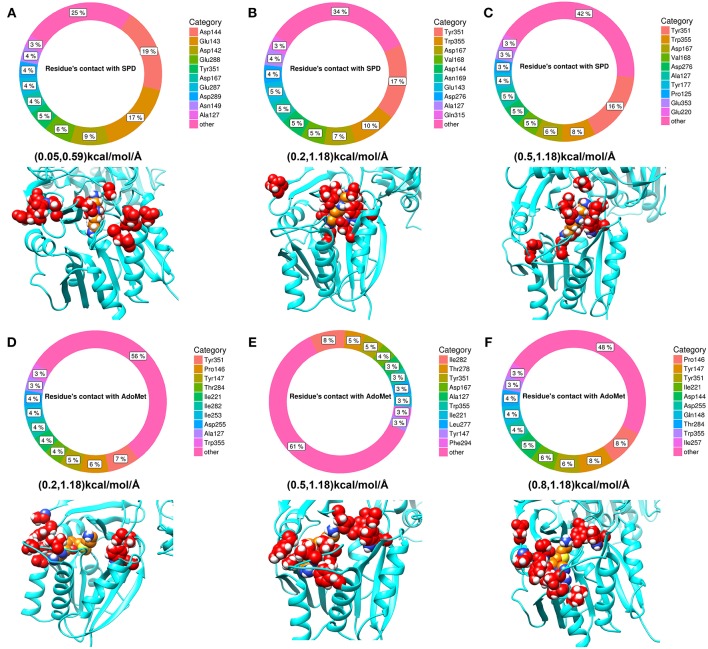
The top 10 residues with most contacts with substrates. The percentage indicates the ratio of contacts contributed by individual residues. **(A–C)** Show the top 10 residues with most contacts with spermidine under different force ranges shown in parentheses (see Method section for details). These residues are highlighted in red in the structure and substrate is colored in orange. **(D–F)** show the top 10 residues with most contacts with AdoMet under different force ranges. These residues are highlighted in red in the structure while substrate is colored in orange.

Since the ligands in the experimental structure are buried, there is no clear indicator how the ligands get inside the corresponding pocket. Here, we applied DFMD to model the association of both spermidine and AdoMet with SpmSyn. We speculate that SpmSyn residues experiencing frequent contacts with the ligands will be within the binding pocket or its “entrance.” Thus, we carried 10 independent DFMD runs and recorded contacts between the substrate and non-hydrogen atoms of SpmSyn using 4 Å cut-off. To see the sensitivity of the results with respect to the parameters of simulations, three ranges of parameters were used (see Method section for details). The total contacts for each residue during 10 independent simulations were calculated and the top 10 residues are shown in Figure 5. One can see that these residues are clustered in space and either form the binding pocket or are on SpmSyn surface close to the binding pocket. These surface residues are the entrance of the corresponding binding pocket. The critical role of some of these residues was previously demonstrated experimentally, indicating that if mutated, the activity of SpmSyn dramatically decreases (Wu et al., 2008).

#### Conformational Changes Induced by the Binding

**Figure 6:**
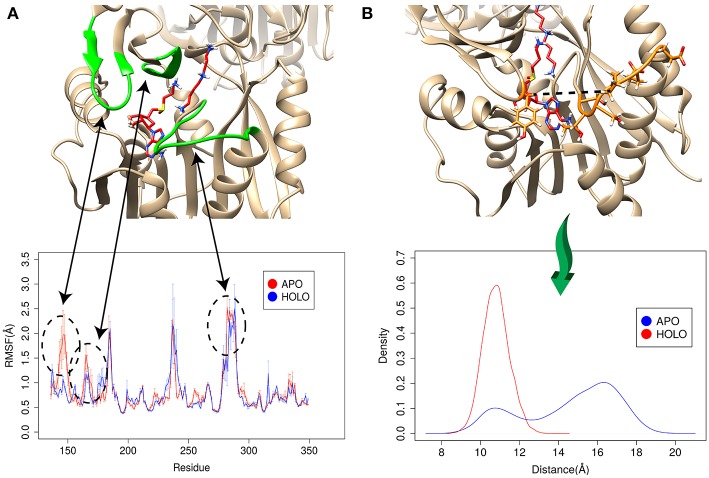
Simulations for APO and HOLO spermine synthase. **(A)** RMSF for APO and HOLO enzyme. The regions reveal large flexibility changes upon substrate binding are highlighted with green. **(B)** Quantitively evaluation of the conformational changes upon ligand binding by comparing the distance between CA of Tyr147 and mean CA positions of residues 280–285 (shown with dashed line in panel B). The same panel shows the distribution of the distance for APO and HOLO SpmSyn.

The enzyme-substrate binding is typically referred to follow either “lock and key” of “induced-fit” (“conformational selection”) models (Benkovic and Hammes-Schiffer, 2003; Johnson, 2008) or something in between (Tajielyato et al., 2018). Since there is not clear entrance to any of the corresponding binding pockets, the SpmSyn substrate binding should be following induced-fit mechanism (especially for AdoMet binding, which is indeed deeply buried). To identify plausible conformation changes induced by the substrate binding, we first carried out two independent 50 ns simulations for both APO (substrate free state) and HOLO (substrates bound state) SpmSyn homo-dimer. During the simulations, we calculated the root mean square fluctuation (RMSF) to reveal conformational changes (Figure 6A). Upon AdoMet and spermidine binding, three loop regions (residues 140–145, 162–166, and 280–285) as shown in Figure 6 are stabilized and are much less flexible compared with APO structure. Such observations indicate that the binding pockets and their “entrances” of spermine synthase probably adopt different conformations in bound and unbound state. In case of AdoMet, we identify several residues, Gln150, Glu222, Ile284, and Ser285, which make H-bonds with AdoMet upon binding. To evaluate the conformational changes induced by AdoMet binding, we calculated the distance between CA of Tyr147 and mean CA positions of residues 280–285 (colored as orange in Figure 6B) over the simulation time. The resulting density plot of the distance distribution for APO and HOLO SpmSyn (Figure 6B) indicates that there is one well-defined conformational state in HOLO SpmSyn. In APO state, there are two conformations: one with relatively high population and different from the HOLO conformation, and another similar to HOLO conformation but having low population. Thus, prior to the binding, two conformational states (distance around 11 Å and distance around 16 Å) are observed, which corresponds to open and closed conformations of the AdoMet binding pocket. Upon AdoMet binding, the binding pocket is stabilized and locked into closed conformation (distance around 11 Å). This observation speaks in favor of the binding mechanism that result in a shift of the populations of existing conformational states.

#### Order of Substrates Binding

**Figure 7:**
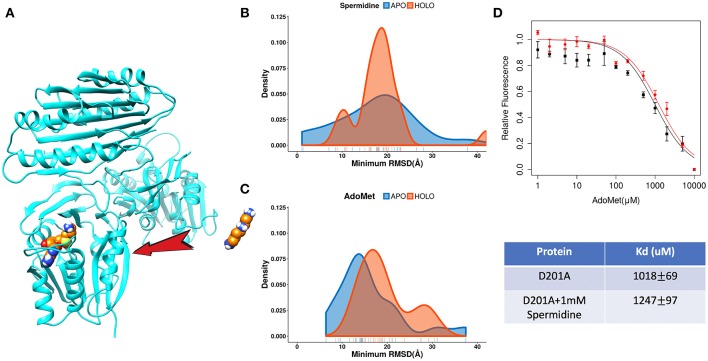
The competitive inhibition of substrates in Spermine synthases. **(A)** The set-up of the DFMD simulation, where one substrate was bound in the pocket and another substrate was steered. **(B)** Comparison of the density map (minimum RMSD) for spermidine in case of AdoMet already bound. **(C)** Comparison of the density map (minimum RMSD) for AdoMet in case of spermidine already bound. **(D)** Experimentally measured relative florescence of AdoMet binding to D201A spermine synthase with (red color) and without (black color) the presence of spermidine and the corresponding dissociation constant (Kd).

The catalytic reaction of spermine synthase requires one aminopropyl group of AdoMet to be cleaved and then added to spermidine resulting in spermine (Pegg and Michael, 2010). There are two plausible scenarios for substrate binding: (a) substrates bind independently of each other or (b) they bind sequentially. To investigate which scenario is more likely, we carried out DFMD simulations such that we kept one substrate in the binding pocket while steered another substrate (Figure 7A). The results indicate that the success rate (low RMSD) is reduced for both binding substrates if the other one is already bound (Figures 7B,C). In another words, binding of one of substrates inhibits the binding of the second one. To explain such inhibition effects, we performed structural and electrostatic potential analysis. In terms of AdoMet binding, our previous finding indicates that loops near the binding pocket are locked into closed conformation upon AdoMet binding. Structural analysis indicates that these loop regions are rich of negatively charged residues. As the spermine is highly positively charged (+3) and in closed conformation these loop regions are accessible, the spermidine recognizes these negative charged residues and binds there instead of the correct binding pocket. However, while the distribution is shifted to the right (larger RMSD, Figure 7B), still the spermidine can successfully bind in presence of AdoMet. Thus, the presence of AdoMet does not affect spermidine binding by much. In reverse, if spermidine binds first, it reduces the negative potential due to its +3e net charge. This reduces the electrostatic guidance of AdoMet, resulting in less effective binding (Figure 7C). Relative florescence experiments were performed to validate the computational predictions. We selected a mutation D201A, which is experimentally known to switch off the catalytic reaction in spermine synthase (Wu et al., 2008) and thus we can measure the binding affinity of one substrate in presence of another substrate. As shown from the florescence measurement (Figure 7D), a decreased binding affinity for AdoMet was observed when adding 1 mM spermidine, which indicate partial inhibition by spermidine (Figure 7D, the Table). These experimental observations indirectly support computational findings presented in this work.

## Conclusion

DFMD is a very fast and effective approach for modeling protein-ligand interactions involving long-range electrostatic interactions. While in this work we demonstrate it using DelPhiForce and NAMD, the same approach can be easily modified to any other MD package. DFMD can be downloaded from http://compbio.clemson.edu/downloadDir/DelphiForce_MD.tar.

DFMD offers three options for providing guiding forces to the corresponding stetted MD package and selecting the most suitable option is up to the users. Here we outline some recommendations to help users choice the option according to the goals of their investigations. Thus, if one wants to find plausible binding trajectories and the entrance of the binding pocket(s) in quick and affective manner, the best choice is accelerated modeling (option “a”), such that the atomic DelPhiForces are calculated constantly until the end of simulation. If one wants to deliver predictions of the binding mode, in addition to the binding trajectories and entrance of the binding pocket, then option “b,” i.e., the cut-off modeling, such that the atomic DelPhiForces are calculated only if all ligand atoms are farther away from the receptor than the cut-offs in MD simulations, should be selected. Finally, if one is concerned about double-counting of electrostatic forces, the scaled modeling (option “c”), such that the atomic DelPhiForces are corrected for the forces calculated via GB, should be selected. However, this will result is relatively slow and, in many cases, non-successful binding.

It should be clarified that the DFMD is most efficient in cases involving a ligand that has net charge and cases for which electrostatics is the major driving force for the binding. However, even in cases of a ligand that does not have net charge, the electrostatic forces on individual ligand's atoms may be still important contributors for the binding and thus the DFMD still may have advantages over standard MD simulations. At the same time, many receptor-ligand bindings may not be facilitated by electrostatics, and thus the DFMD is not applicable for such cases. Instead, the DFMD should be applied to cases for which electrostatics is the major driving force of the association.

## Materials and Methods

### DelPhi

Delphi is a Poisson-Boltzmann equation (PBE) solver that uses finite-difference technique to deliver electrostatic potential distribution. Detalis are provided in the corresponding references (Li C. et al., 2012, 2013; Li L. et al., 2012; Jia et al., 2017).

### DelPhiForce

DelPhiForce is a tool within DelPhi distribution package that allows the electrostatic forces to be calculated between source set of atoms (for example atoms of a receptor) and target set of atoms (for example the atoms of a ligand). The forces are calculated via PBE taking into account water phase and presence of mobile ions. Thus, the receptor's atoms are charged according to user-selected force filed parameters, while the ligand's atoms are kept neutral (no charges). The ligand's atoms are kept neutral to avoid the unwanted effect of self-charges. Thus, using finite-difference approach to solve PBE, DelPhi computes the electrostatic potential distribution including at each ligand's atom due to the changes of the receptor. In the default setup, the scale is setup to be 2 grids/A, thus in case of ligand positioned 60 A away from the receptor and percent filling of 70%, this will result is a mesh about 300 × 300 × 300. Note that the computational box includes two molecules, the charged receptor and the uncharged ligand. Then, the gradient of the potential is numerically obtained and multiplied by the known partial charges of the ligand's atoms, resulting in the electrostatic force at each ligand's atom. More details are given in the corresponding references (Li et al., 2017a,b).

### Steered Molecular Dynamics (MD) Simulations

The steered molecular dynamics simulations were performed using NAMD 2.12 (Phillips et al., 2005). For illustration of the method, the structure used for the simulations is the crystal structure of human spermine synthase (SpmSyn) in complex with spermidine and 5-methylthioadenosine (for comparison purposes, since the 5-methylthioadenosine structure is similar to AdoMet, we aligned the AdoMet to the 5-methylthioadenosine and then replaced the 5-methylthioadenosine with AdoMet in the initial structure) (PDB:3C6K) (Wu et al., 2008). Spermine synthase homo-dimer (chain C and chain D) was extracted from the PDB and missing heavy atoms and short loops were fixed before simulations. Amber ff14SB (Maier et al., 2015) force field was used in the simulation and all parameters files for the simulation were prepared with Amber14 tools. Simulations were done with Generalized Born implicit solvent model (GBIS) with ion concentration 0.15 M and solvent dielectric constant 80. The temperature of system was maintained at 300 K using a Langevin thermostat. van der Walls (vdW) and electrostatic interactions were truncated at 18 Å with a switching function from 16 Å (in parallel as indicated in the text simulations were done with cut-off of 120 Å). Periodic boundary conditions were applied in the simulations with a 150 Å cubic box. Constant pulling forces were applied in the steered MD simulation on each atom of the ligand and the direction and the magnitude of the steered forces were calculated with DelphiForce program (Li et al., 2017a,b). The steered forces were recalculated and updated every 500 steps of the simulations.

### Parameters of DFMD Simulations

In the set-up of the DFMD simulations, there are several parameters affecting the performance of DFMD. Below we describe these parameters (see Supplementary Material for more details).

1. *Number of steps for each cycle of updating the steered forces*. In DFMD, the steered forces are recalculated and updated for every cycle of short simulation steps. The most rigorous approach would be to recalculate forces ever step and use some type of running averaging to remove noises in the steering forces. However, this would lead to extremely slow simulations. In DFMD, the MD simulation cycle is set to 500 steps, which generally results in good performance and reasonable computing time. Users are given the option to adjust this parameter.
2. *Steered electrostatic force range (F*_*lower*_, *F*_*upper*_*)*. When the target is away from the receptor, the computed electrostatic forces can be too small to be read by NAMD (Phillips et al., 2005). In the steered MD simulation with NAMD (Phillips et al., 2005), the minimal input steered force is 0.01 kcal/mol/Å (0.7 pN). Thus, if the computed force is smaller than 0.01 kcal/mol/A it will not be taken account into MD simulations. To overcome this limitation, we provide an option for the users to set up the minimal force (*F*_*lower*_). This parameter is used in the following manner: Consider a ligand with N atoms. If the largest computed force among these N atoms (*F*_max_) is less than the *F*_*lower*_ threshold, the forces on all atoms are multiplied by the factor of $\frac{F_{lower}}{F_{\max}}$ in order to reach the user-defined lower boundary. Upon the ligands getting contacts with proteins, the computed electrostatic forces can be large (for example 10 kcal/mol/Å). As the steered forces are not recalculated for every MD timestep, such large steered forces may bring the ligand atoms too close to the receptor causing vdW clashes. To resolve this problem and avoid large overestimation of the forces, we provide another parameter, the upper boundary of input steered forces (*F*_*upper*_). When the maximum calculated electrostatic forces (*F*_max_) exceed the *F*_*upper*_, all atomic forces are reduced by the factor of $\frac{F_{upper}}{F_{\max}}$ in order to make them not larger than the *F*_*upper*_. The test of different combinations of steered electrostatic force range (*F*_*lower*_, *F*_*upper*_) is presented in the Supplementary Material.
3. *Diffusion constant of the simulations*. It is well-known that diffusion plays critical role in many biological systems (Zhou, 1998; Schlichting, 2000; Gabdoulline and Wade, 2002). In the simulation, Langevin dynamics is applied for the modeling of dynamics, where the diffusion effects are simulated via random applied forces and velocity dependent frictions. In the NAMD simulations, the Langevin damping coefficient is mainly used to manipulate the diffusions in the simulations, where small damping values generally result in more intensive diffusion. Thus, a proper selection of damping coefficient is essential for correct modeling of the diffusion. We provide users with an option to adjust the damping coefficient if needed.
4. *Initial position of targets in the simulation set-up*. The starting steered forces are determined by the initial position and orientation of the molecules in the simulation set-up. It is emphasized that the ligand should be positioned far away from the receptor to allow the long-range electrostatic forces to guide the ligand to the correct binding spot.

### Electrostatics Forces and Potential Calculation

The electrostatic forces applied in the steered MD simulation were computed using DelphiForce program (Li et al., 2017a,b), which is a tool to calculate the electrostatic forces at atomic level. The electrostatic potential calculations were performed with Delphi program (Li L. et al., 2012, 2013). The dielectric constant for protein and solvent was 2 and 80, respectively, and the salt concentration was set to 0.15 M. The percentage filling of the box was 70 with the scale of 1 grid/Å.

### Amber Force Parameter Files for Substrates AdoMet and Spermidine

The force-field parameters and atomic charges of substrates AdoMet and Spermine are needed to carry out the simulations. First, to determine the atomic charges, geometry optimization and electrostatic potential (ESP) calculations for both ligands were performed using Gaussian03 program. The Gaussian output was then read by the antechamber and then the restrained electrostatic potential (RESP) charges (Bayly et al., 1993) were computed based on the determined ESP. The final determined charges and the geometry optimized structure are used to generate the prep topology files for both ligands. Force-field parameters of both ligands are taken from the general Amber force field (Wang et al., 2004) (gaff). Force-field parameters missing in gaff are determined with the parmchk tool of AMBER to generate the frcmod files.

### Molecular Dynamics Simulation for APO and HOLO Spermine Synthase

The structure used for simulation is crystal structure of human spermine synthase in complex with spermidine and 5-methylthioadenosine (PDB:3C6K) (Wu et al., 2008) The original PDB structure consists of two spermine synthase dimer copies and we took the dimer (chain C and chain D) which has the least missing loops and heavy atoms for our study. The extracted dimer was subjected to Profix to fix missing atoms and loops. MD simulations were performed with NAMD 2.12 using Amber ff14SB (Maier et al., 2015) force field and the input files for the simulation are prepared with Amber14 tools. The hydrogens of protein were added using the Reduce program and then LEaP is used to generate the topology and parameters for the simulation. Proteins were solvated using TIP3P watermodel in cubic water box with at least 12 Å from the protein to the edge of box. The ion concentration was maintained at 0.15 M and the net charge of system were neutralized by adding 125 Na+ and 93 Cl- ions. Langevin dynamics with periodic boundary conditions were applied in the simulation. VDW and electrostatic interactions were truncated at 12 Å with a switching function from 10 Å. Particle Mesh Ewald (PME) was applied for long-range electrostatic interaction calculations. First, the system underwent a 5,000-step minimization with a fixed backbone, and then a subsequent 5,000-step minimization without constraint. Then, all atoms in the protein were fixed for 100 ps equilibration of the water. Harmonic constraint of 1 kcal·mol^−1^·Å^−2^ was applied to the protein alpha carbon atoms (CA), and the system was then gradually heated from 0 K to 310 K with 1,000-step/K in the NVT simulation. The system was maintained at 310 K for 1 ns equilibration with CA constraints and another 2 ns equilibration without constraints in NVT system. Finally, the system was switched to an NPT simulation and all constraints were removed for the 50 ns production run.

### Expression and Purification of Human Spermine Synthase

The expression construct of Spermine Synthase was firstly reported in 2008 (Wu et al., 2008) and was kindly supplied by Dr. Hugo Sanabria at Clemson University. The plasmid with the wild-type human SpmSyn gene was used as the template for the construction of G56S mutant. Overlapping extension PCR procedures were performed as previously described by using primers carrying the desired mutation (Yang et al., 2016). The sequences of all two constructs were confirmed by DNA sequencing. All human SpmSyn proteins were expressed in the *E. coli* Rosetta 2 strain by incubating overnight at 14°C with 1 mM isopropyl 1-thio-β-d-galactopyranoside and purified following similar procedures as previously described (Wu et al., 2008).

### Fluorometric Titration Assay

The binding of human SpmSyn to spermidine or AdoMet was analyzed by intrinsic fluorescence measurements. All reagent used in the experiments were freshly prepared. The assays were performed using BioTek Synergy H1 Hybrid Reader. Various amounts of spermidine or AdoMet were added to 200 μl of 50 mM HEPEs buffer (pH 7.4) containing 1 μM human SpmSyn or with additional 1 mM spermidine. The intrinsic protein fluorescence was measured by exciting the sample at 280 nm and reading the emission at 335–345 nm. All samples were allowed to equilibrate in solution for 5 min, after which the fluorescence was measured at 25°C. Fluorescence values were corrected for inner filter effect and the ligands signal using the same sample mixtures excluding human SpmSyn. The dissociation constant (*K*_*d*_, in unit of molarity) were determined by fitting the fluorescence data according to Equations (1, 2),

(1) $$\begin{array}{r} F_{c}=f_{p}\left(P_{t}-P_{b}\right)+f_{pb}P_{b} \end{array}$$

(2) $$\begin{array}{r} P_{b}=\frac{\left(K_{d}+P_{t}+L_{t}\right)-\sqrt{{\left(K_{d}+P_{t}+L_{t}\right)}^{2}-4P_{t}L_{t}}}{2} \end{array}$$

where *F*_*c*_ represents the corrected fluorescence, *f*_*P*_ is the fluorescence coefficient of free human SpmSyn, *f*_*pb*_ is the fluorescence coefficient of human SpmSyn bound to AdoMet, *P*_*t*_ is the initial total human SpmSyn concentration, and *P*_*b*_ is the concentration of human SpmSyn bound to AdoMet. *L*_*t*_ is the total AdoMet concentration in the binding solution. Each concentration was averaged from at least three independent repeats.

## Data Availability

The datasets generated for this study are available on request to the corresponding author.

## Author Contributions

YP carried the computational research and developed DFMD. YY carried the experiments. LL developed DelPhiForce. ZJ contributed to the calculations. WC and EA supervised the investigations. YP, YY, and EA wrote the manuscript.

### Conflict of Interest Statement

The authors declare that the research was conducted in the absence of any commercial or financial relationships that could be construed as a potential conflict of interest.

## Supplementary Material

Supplementary Video 1 100 ns GBIS MD simulations using standard cut-off of 18 Å to model spermidine binding to SpmSyn.

Supplementary Video 2 100 ns GBIS MD simulations using cut-off of 120 Å to model spermidine binding to SpmSyn.

Supplementary Video 3 1 ns DFMD simulations to model spermidine binding to SpmSyn.
