# Erratum: DFMD: Fast and Effective DelPhiForce Steered Molecular Dynamics Approach to Model Ligand Approach Toward a Receptor: Application to Spermine Synthase Enzyme

**DOI:** 10.3389/fmolb.2019.00111

**Published:** 2019-10-29

**Authors:** Frontiers Production Office

**Affiliations:** Frontiers Media SA, Lausanne, Switzerland

**Keywords:** electrostatic interaction, steered molecular dynamics simulation, protein-ligand binding, spermine synthase, electrostatic funnel, electrostatic forces

Due to a production error, the link in the first paragraph of the Conclusion was incorrectly given as: “http://compbio.clemson.edu/downloadDir/delphiforce.tar.gz.” The correct link is: http://compbio.clemson.edu/downloadDir/DelphiForce_MD.tar.

The publisher apologizes for this mistake. The original article has been updated.

