# Supplementary material for: DFMD: Fast and Effective DelPhiForce Steered Molecular Dynamics Approach to Model Ligand Approach Toward a Receptor: Application to Spermine Synthase Enzyme
Source: Front Mol Biosci. 2019 Sep 4;6:74. doi: 10.3389/fmolb.2019.00074 (PMC6737077; doi:10.3389/fmolb.2019.00074)
Supplement: Supplementary file 1 [file Table_1.DOCX]

Supplementary Material

**DFMD: Fast and effective DelPhiForce steered molecular dynamics approach to model ligand approach toward a receptor: Application to spermine synthase enzyme.**

Yunhui Peng^1^, Ye Yang^2^, Lin Li^3^, Zhe Jia^1^, Weiguo Cao^2^, and Emil Alexov^1^ *,

^1^  Computational Biophysics and Bioinformatics Lab, Department of Physics, Clemson University, Clemson, SC 29634, USA;

^2^  Department of Genetics and Biochemistry, Clemson University, Clemson, SC 29634, USA;

^3^  Computational Biophysics and Bioinformatics Lab, Department of Physics, University of Texas at El

Paso, El Paso, TX 79968, USA

* Correspondence: [ealexov@clemson.edu](mailto:ealexov@clemson.edu); Tel.: +864-908-4796; Fax: +864-656-0805


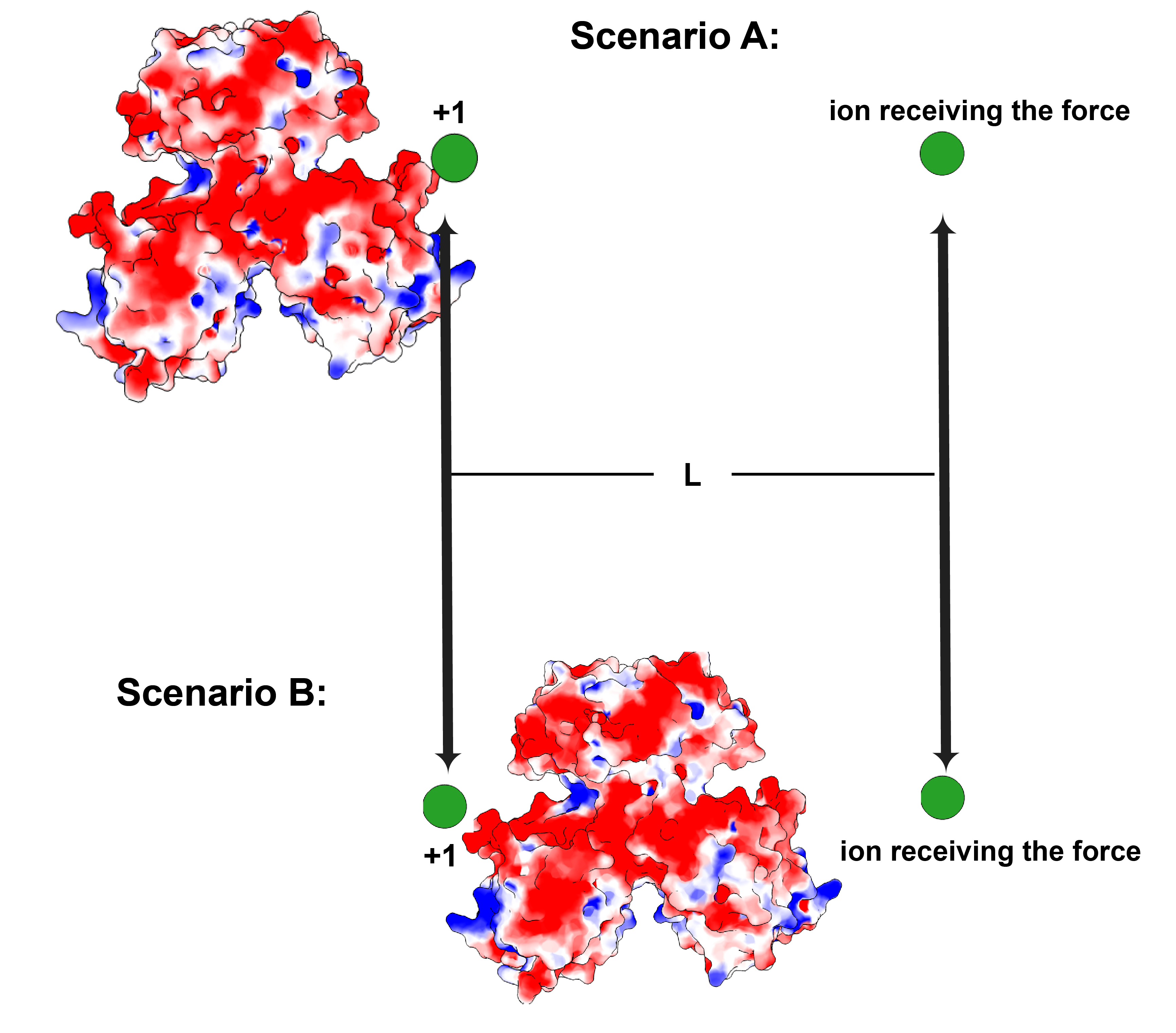


Figure S1: Two scenarios of one charged ion (+1e charge) situated at two equidistant positions from another ion (ion receiving the force): (scenario A) the charged ion is on the right side of the protein, (scenario B) the protein is situated between the ions (charged ion and the ion receiving the force).

|  | NAMD | | | DelphiForce | | |
| --- | --- | --- | --- | --- | --- | --- |
|  | Fx | Fy | Fz | Fx | Fy | Fz |
| Scenario A | 4.35e-4 | -2.25e-5 | 1.04e-5 | 8.29e-4 | 0 | -2.04e-2 |
| Scenario B | 4.34e-4 | -2.17e-5 | 1.19e-5 | 4.74e-4 | 0 | 0 |

Table S1: Comparison of the long-range electrostatic forces in NAMD GBIS model and DelphiForce at two scenarios. The force between two ions (+1 charge) are computed at two different positions as described in Figure S1. Each atomic force has three components (Fx, Fy and Fz), in units kcal/mol/Å.

**
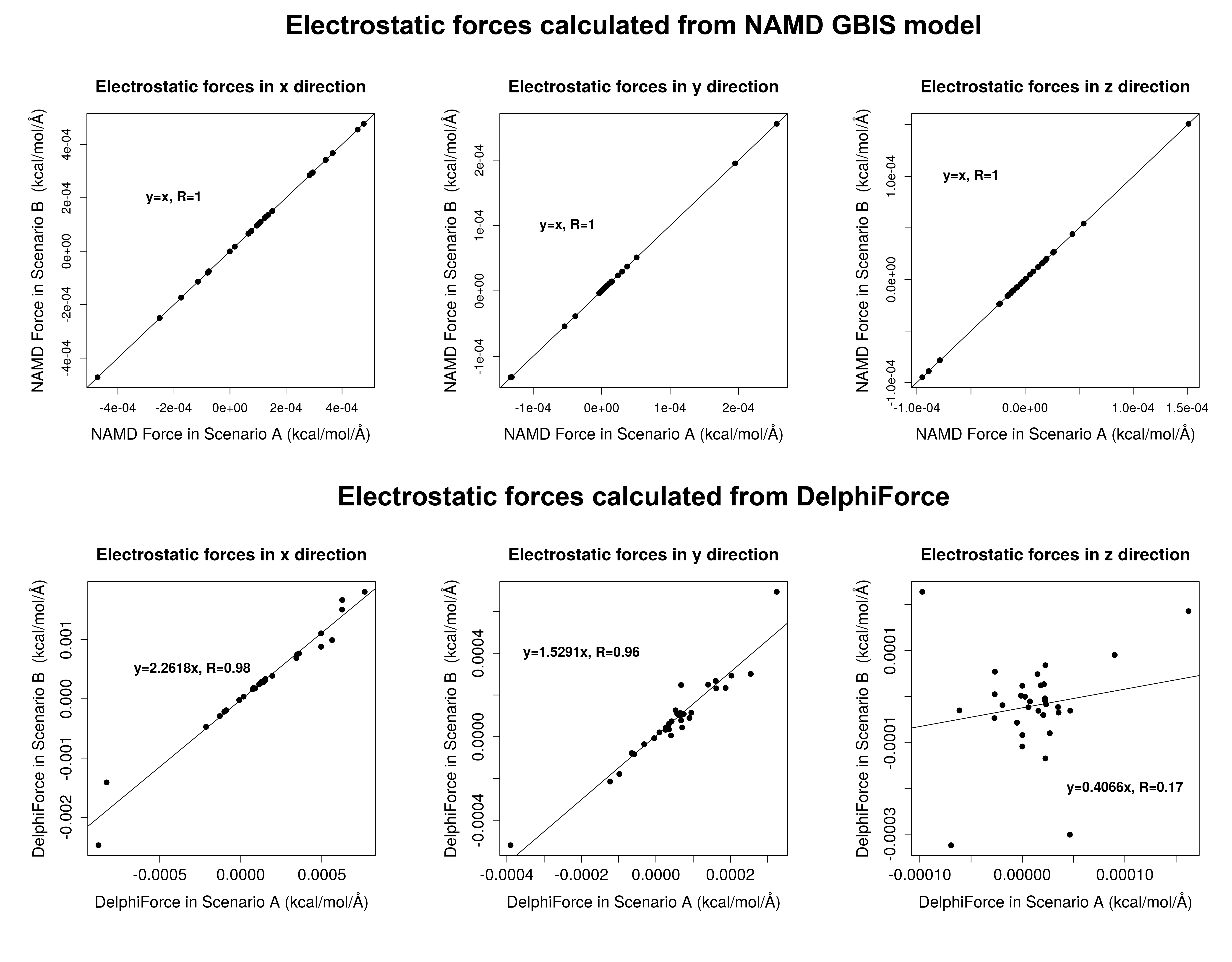
**

Figure S2: Comparison of the long-range electrostatic forces in NAMD GBIS model and DelphiForce at two scenarios (Figure S1, where the receiving ion is replaced by spermidine ligand). The ligand has 32 atoms including hydrogens, and thus 32 atomic forces were calculated. Each atomic force has three components (Fx, Fy and Fz), in units kcal/mol/Å.


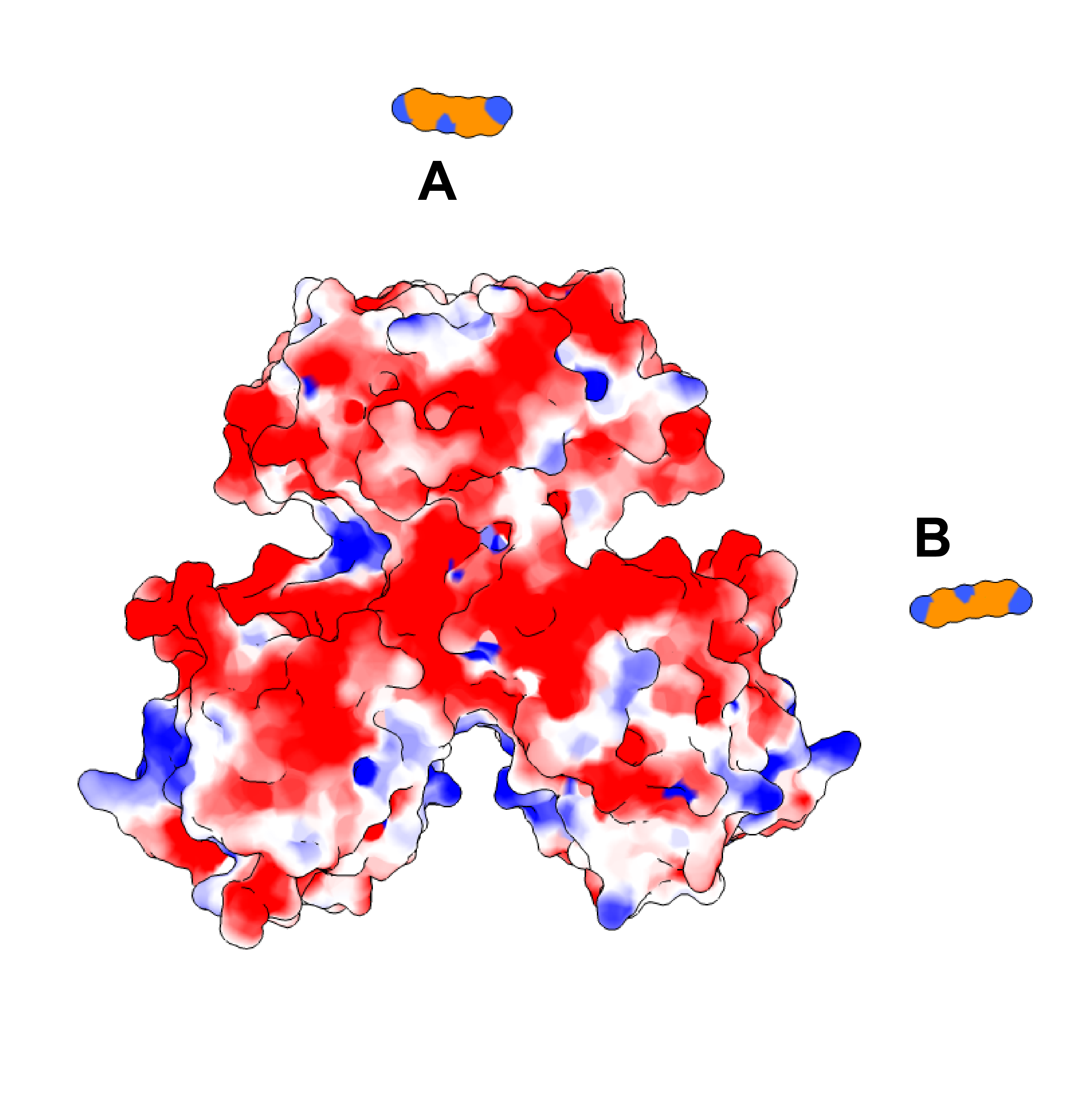


Figure S3: Spermidine positioned 60Å away from the geometrical center of SpmSyn at position A and B, respectively.

**
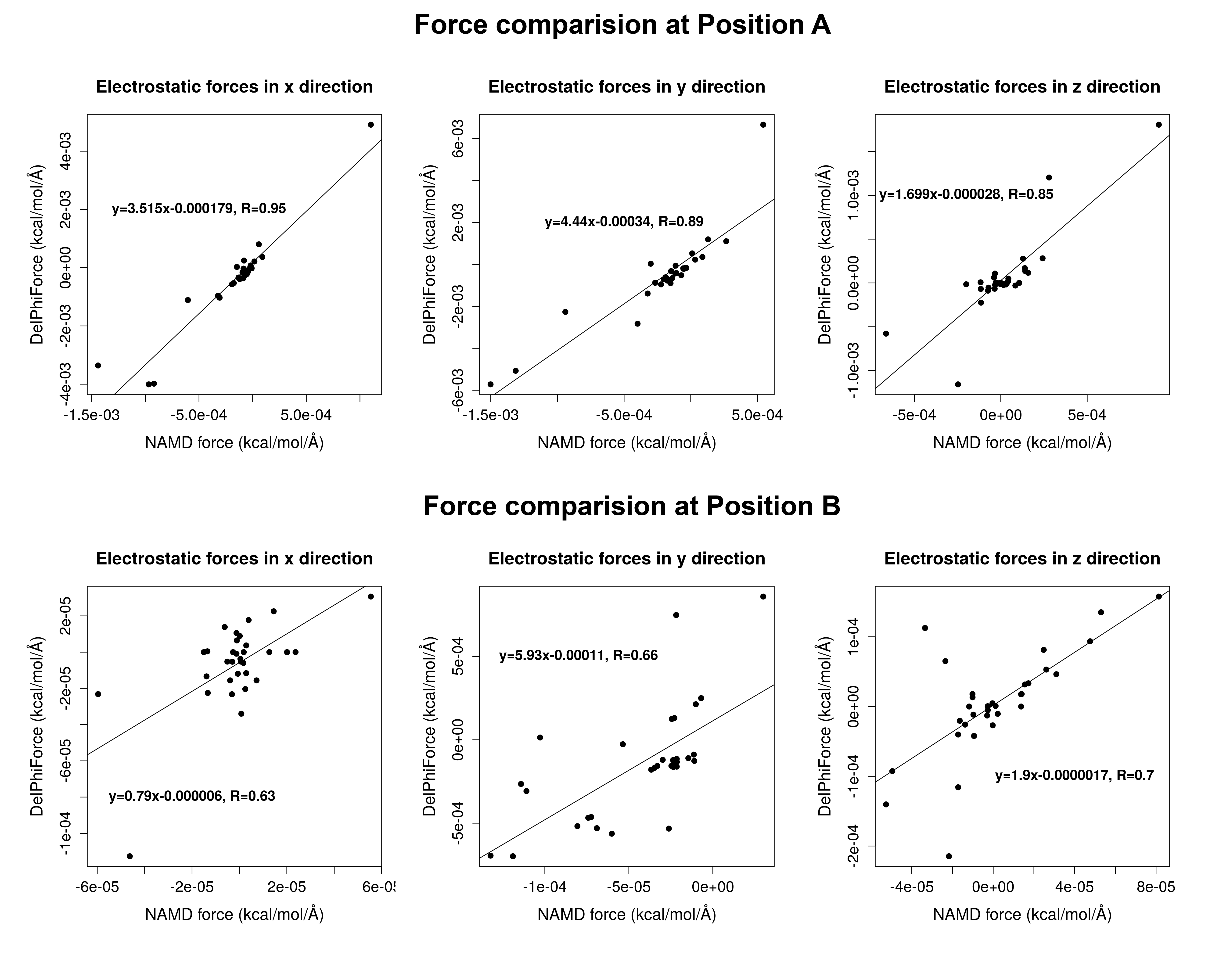
**

Figure S4: Comparison of the long-range electrostatic forces in NAMD GBIS model and DelPhiForce. The force between ligand (spermidine) and protein are computed at two different positions (Figure S3) and their components compared. The slope of the fitting line indicates the overall difference of the magnitude of the atomic forces calculated with GBIS NAMD and DelPhiForce. The DelPhiForce results is some cases are almost 3-4 times larger than those of GBIS NAMD.

***Testing of parameters in DFMD simulation***

The values of the parameters in DFMD simulations described in the main body of the manuscript are expected to affect the simulation performance. Thus, we carried out a test to investigate how these parameters may affect simulation results using a receptor (spermine synthase). The first set of parameters that were tested are the steered electrostatic force range ($F_{lower}, F_{upper}$). We carried out ten independent DFMD simulations for various force ranges. The RMSD of the ligands in the simulation are calculated (as shown in Figure 1d) and the minimal RMSD values from each independent run (representing the closest distance that ligand gets to the binding pocket) are retrieved to draw the density maps for each selected force range (Figure S5). The lower force boundary range was gradually increased with upper force boundary set to 0.59 kcal/mol/Å and 1.18 kcal/mol/Å. For both substrates, a similar tendency was observed that the probability of ligands getting into the binding pockets increases by selecting larger upper and lower steered force boundaries. In addition, as the AdoMet (+1) carries much less charges comparing with spermidine (+3), a larger force ranges are required for successful binding. However; depending on the systems and problem to be investigated, one may have to adjust the range. In this study, we selected (0.05,0.59) and (0.2,1.18) kcal/mol/Å as reference forces ranges for the spermidine and AdoMet, respectively.

**
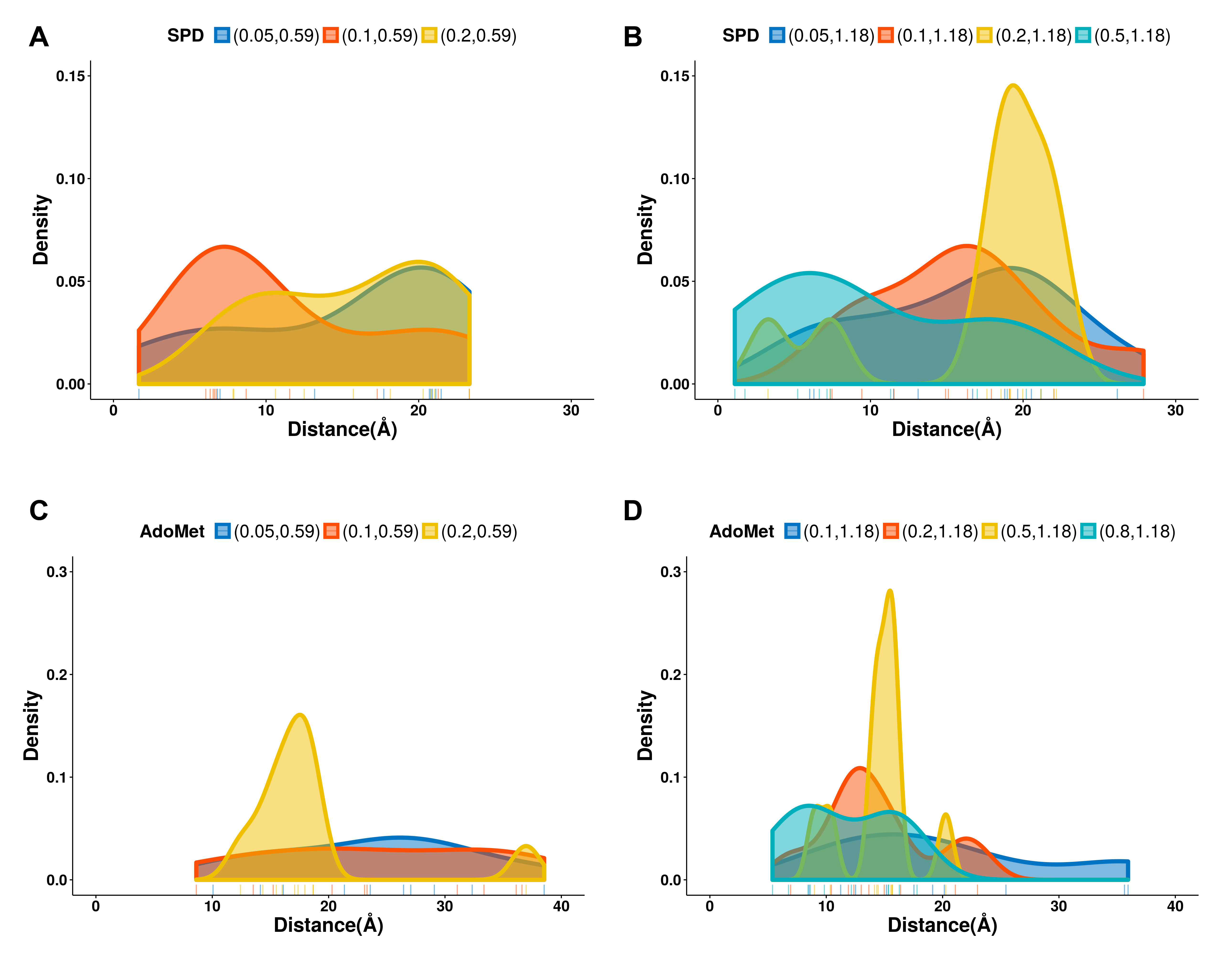
**

Figure S5: DFMD results for AdoMet and Spermidine using different selection of steered force range. The units for force range are in kcal/mol/Å.

Another factor we are interested in is how the initial positions and orientations of the ligands may affect the DFMD simulation results. First, we tested three different initial orientations, where the ligand is rotated along X, Y or Z axis for 90 degrees (Figure S6A), and then 10 independent runs were performed for each orientation. The RMSD of ligand was computed to derive the density map for each orientation of both spermidine and AdoMet (Figure S6B and S6C). It was found that AdoMet is more sensitive to the initial orientations comparing to spermidine. This is expected based on our electrostatic potential analysis (Figure S6B and C), where the charges are homogeneously distributed for the spermidine while AdoMet potential is inhomogeneous. Generally, an initial orientation which provides largest attractive forces should be selected in the simulation to accelerate the binding.

***
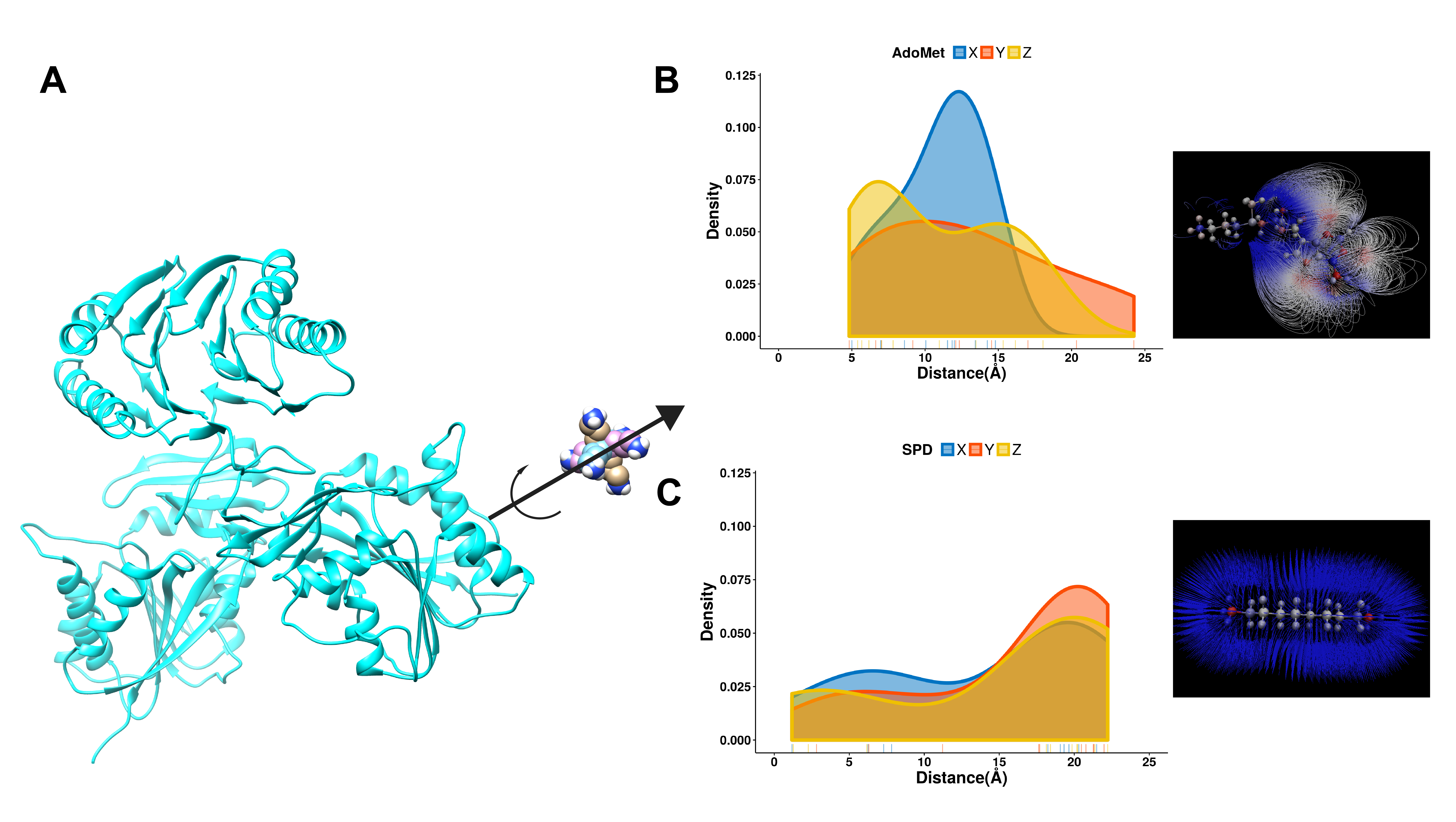
***

Figure S6: DFMD results for AdoMet and spermidine using different initial orientations. The units for force range are in kcal/mol/Å. (A) Three different initial orientations, where the ligand is rotated along X, Y or Z axis for 90 degrees. (B) and (C) Minimum RMSD density map for AdoMet and spermidine at three different initial orientations.
